# Much ‘tattoo’ about nothing; Tattoo pigment mimicking breast microcalcifications on mammography

**DOI:** 10.1016/j.radcr.2021.04.044

**Published:** 2021-05-16

**Authors:** Roisin M. Heaney, Laura Sweeney, Clare Smith, Angela O'Brien

**Affiliations:** Breast Health, Mater Misericordiae University Hospital, 38 Eccles Street, Dublin 7, Ireland

**Keywords:** Microcalcifications, Mammogram artefacts, Digital breast tomosynthesis, Tattoo pigment

## Abstract

Mimics of calcifications on mammography are not uncommon and result in additional investigations that can cause patient anxiety. We describe the case of a 63 year old male who underwent further investigation of calcifications in the superior right breast. Additional imaging and patient examination revealed that the calcifications were located in a color tattoo overlying the medial right pectoralis muscle and actually represented the radio-opaque metallic compounds found in tattoo pigment.

## Introduction

Recall rates for calcifications on mammography have increased in recent years, in part due to the introduction of digital mammography. Recalling a patient for additional mammographic views can cause considerable worry for the patient and every effort is made to reduce artefacts that mimic calcification. We describe a case of skin calcification from a tattoo mimicking microcalcifications within the breast as well as tattoo pigmentation within a contralateral axillary lymph node.

## Case report

A 63 year old male was referred to the symptomatic breast unit with right nipple pain and a retroareolar swelling. There was no personal or family history of breast cancer. The patient had a history of rheumatoid arthritis and dyslipidaemia. His medications were omeprazole, atorvastatin and gabapentin.

**Fig. 1:**
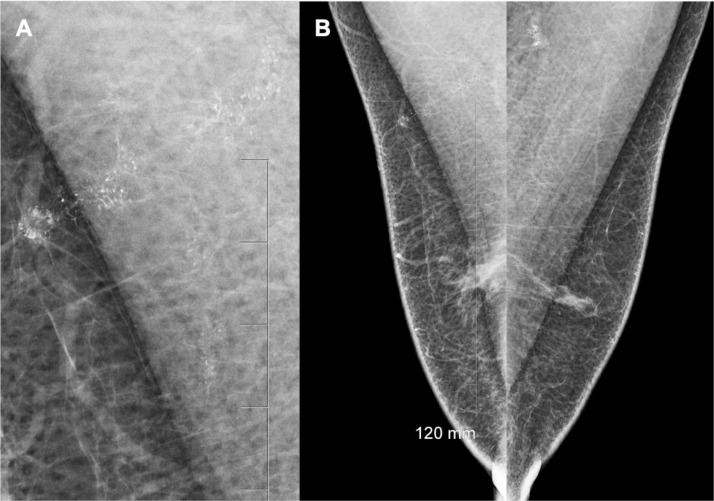
A. Magnified view of the calcifications overlying the right pectoralis muscle. B. MLO views demonstrating the bilateral calcification and mild gynaecomastia.

A standard 2-view digital mammogram was performed which demonstrated mild bilateral gynaecomastia, right greater than left. In addition there were amorphous calcifications in the superior right breast and overlying the right pectoralis major muscle, extending over a distance of 4.5cm. These were only visible on the medial-lateral oblique (MLO) view (Fig. 1). There was a clustered area of similar appearing calcifications overlying the left pectoralis major muscle. Given the bilateral distribution the calcification was thought most likely to represent talc artefact and the patient was recalled for a technical repeat. Despite ensuring that both axillae were cleaned of any possible external artefact, the calcifications persisted on repeat imaging.

**Fig. 2:**
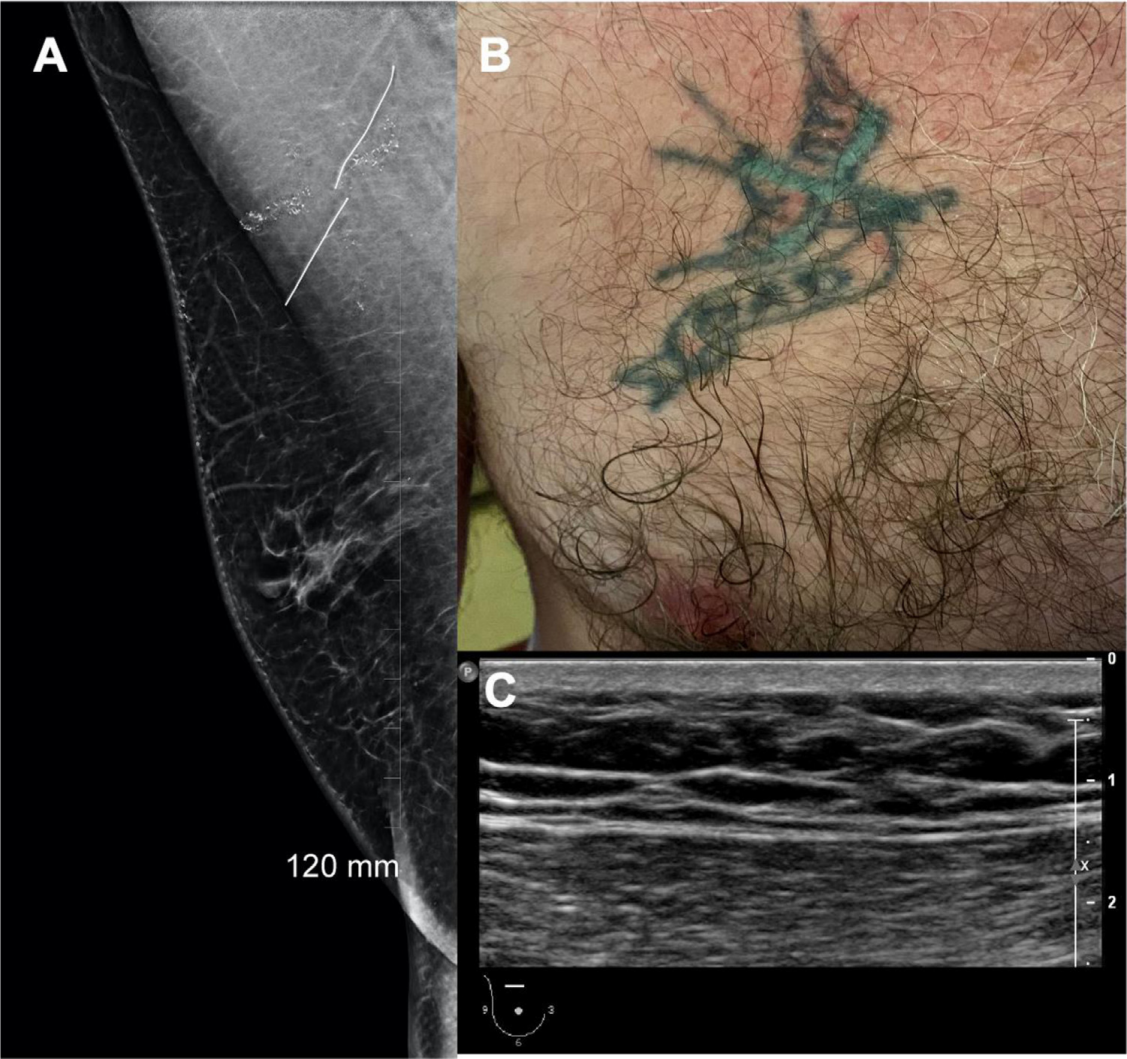
A. Slice 53/74 in the medial breast from the digital tomogram demonstrating the ‘BB’ marker directly overlying the calcifications. B. The corresponding color tattoo. C. Normal ultrasound of the superomedial right breast.

At this point the calcifications were deemed to be concerning for ductal carcinoma in situ (DCIS) and a careful ultrasound of the superior right breast was performed. No abnormality was identified however it was noted that the patient had a color tattoo overlying the medial right pectoralis major muscle (Fig. 2). Two ‘BB’ markers were placed over the tattoo and a tomogram in the MLO position was performed. This revealed the location of the calcifications to be in the medial breast, directly underlying the ‘BB’ markers, corresponding to the site of the tattoo (Fig. 2).

The patient did not have a tattoo on the left chest wall, however there were multiple tattoos on the left arm and forearm. The additional views demonstrated the calcifications to be associated with a nodular density consistent with a lymph node. An ultrasound of the left axilla revealed morphologically normal lymph nodes.

## Discussion

Our case demonstrates two different mammographic artefacts from skin tattoos; calcification of the tattoo pigment within the skin at the site of the tattoo and calcification within an axillary lymph node in the drainage field of a skin tattoo. Our case is only the third reported case of a breast tattoo simulating microcalcifications within the breast, previously described in 1981 [1] and 2005 [2]. In addition, our case is the first case to be demonstrated on tomosynthesis and in a male patient.

Since its introduction, digital breast tomosynthesis has improved the detection of both benign and malignant breast lesions. Initial reports suggested that some calcifications may be missed by tomosynthesis as the conspicuity of the calcifications is affected by the lower spatial resolution of tomosynthesis compared to standard digital mammography [3]. In our experience, breast calcifications tend to be more conspicuous on tomosynthesis however magnification views are used for further assessment of calcifications in our institution. In this case however, tomosynthesis proved to be invaluable in confirming the exact position of the calcifications in the patient's tattoo.

Tattoo pigments contain metallic compounds which, when deposited in the skin, are radio-opaque. A case report in Legal Medicine documents how the radio-opaque tattoo pigments in a deceased person on a post-mortem CT permitted 3-D reconstruction of the original tattoo [4]. Accumulation of tattoo pigments in draining lymph nodes is an uncommon but well documented phenomenon that results in the appearance of calcified lymph nodes on mammography [5,6]. Another more common mimic of calcified lymph nodes on mammography is the presence of talc powder from certain deodorants, antiperspirants, powders and soaps, and patients are asked to avoid applying these on the morning of imaging. The presence of tattoo pigment within lymph nodes also has the potential to mimic sentinel node blue dye in a patient undergoing sentinel node sampling, potentially leading to the resection of additional axillary lymph nodes and the under staging of the patient's breast cancer [7].

Patients undergoing mammography in our institution complete a questionnaire which documents any family history and previous breast intervention, and prominent skin lesions are identified on the mammogram using ‘BB’ markers. Based on our experience from this case we now recommend that upper body and extremity tattoos are documented in this questionnaire.
